# Modelling of a triage scoring tool for SARS-COV-2 PCR testing in health-care workers: data from the first German COVID-19 Testing Unit in Munich

**DOI:** 10.1186/s12879-022-07627-5

**Published:** 2022-08-01

**Authors:** Hannah Tuulikki Hohl, Guenter Froeschl, Michael Hoelscher, Christian Heumann

**Affiliations:** 1 Division of Infectious Diseases and Tropical Medicine, Medical Center of the University of Munich (LMU), Leopoldstr. 5, 80802 Munich, Germany; 2 German Center for Infection Research (DZIF), Partner Site Munich, 80802 Munich, Germany; 3 Department of Statistics, University of Munich (LMU), Ludwigstr. 33, 80539 Munich, Germany

**Keywords:** SARS-COV-2, COVID-19, Triage, Prediction model, Public health, Epidemiology, Munich, Germany

## Abstract

**Background:**

Numerous scoring tools have been developed for assessing the probability of SARS-COV-2 test positivity, though few being suitable or adapted for outpatient triage of health care workers.

**Methods:**

We retrospectively analysed 3069 patient records of health care workers admitted to the COVID-19 Testing Unit of the Ludwig-Maximilians-Universität of Munich between January 27 and September 30, 2020, for real-time polymerase chain reaction analysis of naso- or oropharyngeal swabs. Variables for a multivariable logistic regression model were collected from self-completed case report forms and selected through stepwise backward selection. Internal validation was conducted by bootstrapping. We then created a weighted point-scoring system from logistic regression coefficients.

**Results:**

4076 (97.12%) negative and 121 (2.88%) positive test results were analysed. The majority were young (mean age: 38.0), female (69.8%) and asymptomatic (67.8%). Characteristics that correlated with PCR-positivity included close-contact professions (physicians, nurses, physiotherapists), flu-like symptoms (e.g., fever, rhinorrhoea, headache), abdominal symptoms (nausea/emesis, abdominal pain, diarrhoea), less days since symptom onset, and contact to a SARS-COV-2 positive index-case. Variables selected for the final model included symptoms (fever, cough, abdominal pain, anosmia/ageusia) and exposures (to SARS-COV-positive individuals and, specifically, to positive patients). Internal validation by bootstrapping yielded a corrected Area Under the Receiver Operating Characteristics Curve of 76.43%. We present sensitivity and specificity at different prediction cut-off points. In a subgroup with further workup, asthma seems to have a protective effect with regard to testing result positivity and measured temperature was found to be less predictive than anamnestic fever.

**Conclusions:**

We consider low threshold testing for health care workers a valuable strategy for infection control and are able to provide an easily applicable triage score for the assessment of the probability of infection in health care workers in case of resource scarcity.

**Supplementary Information:**

The online version contains supplementary material available at 10.1186/s12879-022-07627-5.

## Introduction

Health care workers (HCW) play a crucial role in the COVID-pandemic, which has caused by March 2022 over 450 million infections worldwide and taken over 6 million lives [1]. While healthcare workers are at risk of contracting COVID-19 themselves by caring for infected patients, they are also in contact with the most vulnerable segments of society: those who rely on medical assistance. It is therefore crucial to avoid infections among HCWs to avert further SARS-COV-2 infections and -deaths.

In the 1st months of the pandemic HCW in Munich, Germany faced additional challenges: while today effective personal protective equipment is commonly available and community-wide low-threshold testing is being implemented, in the 1st months of 2020 only limited means for diagnostics were available. Urgent need for testing, combined with limited resources, made strict testing triage necessary. Societies with more limited financial and structural resources continue to face this dilemma to this day [2]. With vaccination rates remaining low in many countries worldwide [1] and with the emergence of virus variants that challenge hopes of eradicating the virus in the near future [3], testing is likely to remain a necessity.

Many tools have been developed for screening of suspected COVID cases [4, 5]. These, though, are predominantly based on patient groups from the general population and do not specifically target health care workers. Some screening scores require diagnostics such as extensive laboratory analyses or imaging, making them rather unsuitable for a setting like in-house medical staff screening [6–10]. With comparatively small numbers of outcomes but numerous potential symptoms and prognostic factors being discussed, especially early publications bear the risk of a lack of accuracy [11]. Other points of critique include poor reporting and high risk of bias [4].

With this research we aimed to develop and evaluate an easy-to-use triage scoring tool specifically applicable for health care workers in an outpatient setting. Additionally, we aimed to explore how the correlation of our variables and the SARS-COV-2 testing outcome of patients has changed with a growing data set over time and to provide an explanatory approach as to why publications from early on in the pandemic show different and sometimes contradictory findings regarding COVID-characteristics.

This study is reported according to TRIPOD guidelines for transparent reporting of a multivariable prediction model for individual prognosis or diagnosis [12].

## Methods

### Study design

We conducted a monocentric, retrospective analysis of patient data of the Corona Testing Unit Munich (CTU). All patients admitted between January 27 and September 30, 2020, who could retrospectively be identified as health care workers were included. We defined HCWs as employees at health care providers (hospitals and nursing homes), and patients with a profession in the health sector. Data for repeated testing visits per patient was included. However, data from patients with follow-up testing after an initial positive result were excluded from the analysis. Seven tests produced no viable result and were excluded from analysis.

### Study setting

Our study was conducted at the Corona Testing Unit Munich at the Division of Infectious Diseases and Tropical Medicine of the University Hospital, Ludwig-Maximilians-Universität (LMU) Munich, Germany. In its operating time of January 27, 2020, to September 30, 2020, 5339 patients were tested for SARS-COV-2 in the CTU. Patients were referred by institutions, including the occupational health departments of the LMU Klinikum and 15 other hospitals as well as 17 occupational health physicians responsible for nursing homes in and around Munich, or self-referred. Patients admitted by self-referral were triaged and tested in accordance with guidelines as stipulated by the Robert Koch Institute, the German federal government agency and research institute responsible for disease control and prevention.

Anamnesis of the first patients admitted to the CTU was taken by our team of physicians in a patient interview in order to avoid nosocomial surface contact transmission through paperwork touched by patients. From February 27th on, patients were asked to fill out a structured case report form (CRF). Questions on the CRF included sociodemographic data, workplace and occupation, travel history, specifics to any close contact with confirmed COVID-cases and a list of possible symptoms. Questions about pre-existing conditions were part of early CRF versions but later excluded as patient throughput and hence workload increased. Additionally, vital parameters were collected of some patients in a systematic fashion at the beginning of operations, but later-on suspended.

### Testing and laboratory analysis

Naso- or oropharyngeal swabs were taken by a team of physicians and specially trained medical students. COVID-19 was confirmed using real-time polymerase chain reaction (RT-PCR) by the Institute for Microbiology of the Armed Forces in Munich, the Max-von-Pettenkofer-Institute of the Ludwig-Maximilians-Universität Munich and the private medical laboratory “Labor Becker & Kollegen” in Munich.

### Analysis

We chose STATA (Version 16.1. College Station, TX: StataCorp LLC) for general statistical analysis. Stepwise variable selection and bootstrapping was conducted with package “MASS” (version 7.3–54), package “pROC” (version 1.18.0), and package “GmAMisc” (version 1.2.0), R (version 4.0.4). To represent patient characteristics, possible exposures, and testing outcomes, we used frequencies and percentages for nominal and means and interquartile ranges for numeric variables. In case of patients with multiple tests over time, each testing occasion was analysed independently.

#### Triage scoring model

To explore risk factors associated with PCR test positivity, univariable logistic regression models with a binary test outcome (SARS-COV-2 PCR positive/negative) as the dependent variable were used.

To evaluate a possible impact of recent travels abroad, we additionally analysed the correlation between test-positivity and the national 7-day incidence of confirmed positive cases per 100,000 inhabitants at the reported travel destination at the time of admission [13].

Of the variables with p-values < 0.2 in univariable logistic regression, we selected variables for the multivariable logistic regression model based on stepwise backward selection [14]. Variables for which more than 10% of the data were missing (e.g., pre-existing conditions and vital parameters upon admission) were excluded due to data scarcity. Through complete-case analysis (utilisation only of cases for which there are no missing values on any of the model variables), 3362 observations were included in the final analysis. We assessed the discriminative performance of the final model through the Area Under the Receiver Operating Characteristics Curve (AUC) and its 95% confidence interval.

We used the bootstrap method for internal validation of our final model (repeated 1000 times) and calculated the difference (AUCoriginal − AUCcorrected) to assess the bootstrap-corrected performance of our original model [15].

The coefficient of each covariate of the final model was then converted into a weighted point-scoring system by multiplying by the factor two and mathematical rounding to the next integer.

#### Analysis of variable characteristics over time

To study potential changes in the influence of variables on a model over time, we calculated the odds ratio (OR) and the 95% confidence interval in univariable logistic regressions with cumulative weekly datasets, where the OR was calculated per variable for each week with the sum of all patients up to this point in time.

## Results

### General patient characteristics

In the observed period between January 27 and September 30, 2020, 5339 patients were admitted to the CTU. 3069 patients met the aforementioned criteria and were included in this study. Of the 4197 performed tests, 121 resulted in a COVID-19 diagnosis and 4076 were negative (see Table 1). The mean age of positive patients was slightly higher than that of negative patients (39.9 and 37.9, respectively), as was the percentage of females (73.6% and 69.7%, respectively). In the 43.4% of all patients who stated their profession, nurses (17.8%), physicians (5.8%) and physiotherapists (2.0%) where the most frequent. These professions (plus cleaners) are at the same time overrepresented in the group of positive patients (nurses: 17.7% of negative versus 20.7% of positive patients, physicians: 5.7% versus 6.6%, physiotherapists: 2.0% versus 3.3%, cleaners: 0.7% versus 1.7%), whereas other professions (occupational therapists, researchers, speech therapists, students, trainees and others) were overrepresented in the group of negative patients.

**Table 1:** Sociodemographic information: frequency and percentage by test result

Characteristics	Negative (n = 4076)		Positive (n = 121)		Total (n = 4197)		p value
Age*	37.9	20.0	39.9	21.0	38.0	20.0	0.090
Gender							
Female	2842	69.7%	89	73.6%	2931	69.8%	–
Male	1234	30.3%	32	26.4%	1266	30.2%	0.367
Occupation							
Administration	59	1.4%	1	0.8%	60	1.4%	0.502
Caregiver	27	0.7%	0	0.0%	27	0.6%	NA
Cleaner	30	0.7%	2	1.7%	32	0.8%	0.352
Housekeeping	29	0.7%	0	0.0%	29	0.7%	NA
Medical technical assistant	26	0.6%	0	0.0%	26	0.6%	NA
No information	2299	56.4%	77	63.6%	2376	56.6%	–
Nurse/geriatric nurse/nursing assistant	722	17.7%	25	20.7%	747	17.8%	0.887
Occupational therapist	57	1.4%	0	0.0%	57	1.4%	NA
Other occupation	371	9.1%	3	2.5%	374	8.9%	0.016
Physician	234	5.7%	8	6.6%	242	5.8%	0.957
Physiotherapist	81	2.0%	4	3.3%	85	2.0%	0.460
Researcher/research assistant	31	0.8%	0	0.0%	31	0.7%	NA
Speech therapist	39	1.0%	0	0.0%	39	0.9%	NA
Student	52	1.3%	1	0.8%	53	1.3%	0.585
Trainee	18	0.4%	0	0.0%	18	0.4%	NA

*Age: mean and inter-quartile range. p-value was obtained by univariable logistic regression (NA: p-value calculation not applicable; gender reference “female”, occupation reference “No information”)

COVID-positive patients more frequently presented with symptoms than COVID-negative cases (66.9% versus 29.0%, respectively). Symptomatic COVID-positive patients presented, with a mean of 4.0 days after symptom onset, earlier than symptomatic COVID-negative patients (mean: 5.6 days). The overall most reported symptoms included sore throat (17.2%), cough (15.1%), and rhinorrhoea (14.3%). An overview of all reported symptoms can be seen in Table 2.

**Table 2:** Symptoms: frequency and percentage by test result

Characteristics	Negative (n = 4076)		Positive (n = 121)		Total (n = 4197)		p value
Symptoms							
Asymptomatic	2805	68.8%	39	32.2%	2844	67.8%	–
Symptomatic	1181	29.0%	81	66.9%	1262	30.1%	0.000
No information	90	2.2%	1	0.8%	91	2.2%	NA
Days since symptom onset	5.6	6.0	4.0	3.0	5.5	6.0	0.095
Fever	201	4.9%	39	32.2%	240	5.7%	0.000
Cough	572	14.0%	59	48.8%	631	15.0%	0.000
Shortness of breath	94	2.3%	6	5.0%	100	2.4%	0.067
Sore throat	690	16.9%	33	27.3%	723	17.2%	0.003
Rhinorrhea	545	13.4%	52	43.0%	597	14.2%	0.000
Anosmia/Ageusia	54	1.3%	11	9.1%	65	1.5%	0.000
Sputum production	27	0.7%	6	5.0%	33	0.8%	0.000
Chest pain	34	0.8%	6	5.0%	40	1.0%	0.000
Otalgia	20	0.5%	2	1.7%	22	0.5%	0.076
Wheezing	11	0.3%	1	0.8%	12	0.3%	0.243
Joint pain	36	0.9%	13	10.7%	49	1.2%	0.000
Muscle pain	52	1.3%	15	12.4%	67	1.6%	0.000
Fatigue	94	2.3%	13	10.7%	107	2.5%	0.000
Headache	188	4.6%	22	18.2%	210	5.0%	0.000
Confusion	1	0.0%	0	0.0%	1	0.0%	NA
Seizure	1	0.0%	0	0.0%	1	0.0%	NA
Abdominal pain	11	0.3%	5	4.1%	16	0.4%	0.000
Nausea/Emesis	21	0.5%	4	3.3%	25	0.6%	0.000
Diarrhea	28	0.7%	3	2.5%	31	0.7%	0.023
Conjunctivitis	5	0.1%	0	0.0%	5	0.1%	NA
Eczema	1	0.0%	0	0.0%	1	0.0%	NA
Lymphadenopathy	12	0.3%	3	2.5%	15	0.4%	0.001
Bleeding	2	0.0%	0	0.0%	2	0.0%	NA

*Days since symptom onset: mean and inter-quartile range. p-value was obtained by univariable logistic regression (NA: p-value calculation not applicable). Symptoms were all coded binary (1 = yes, 0 = no), reference is always “no”

Only some patients (n = 356) answered questions regarding pre-existing conditions (see Additional file 1: Table S1). Of these, 34 (10.0%) reported to have been previously diagnosed with asthma, followed by 12 (3.6%) with obesity and 8 (2.4%) with a history of heart disease. Notably, none of the 34 asthma patients received a positive SARS-COV-2 test result.

In a small number of patients, vital parameters were recorded (pulse: n = 162, temperature: n = 166, O2-saturation: n = 159). Of this group, positive patients presented with higher mean pulse (106.0 bpm versus 81.6 bpm) and temperature (37.1 °C versus 36.4 °C) and lower mean O2-saturation (93.0% versus 96.8%).

Table 3 shows a summary of possible exposures: 2756 (65.7%) of all patients reported some sort of contact to a COVID-19 case. At 86.0%, the proportion was even more notable for positive patients. Of the predefined categories, “Colleague” was the most frequently reported (35.7% of negative and 38.8% of positive patients), followed by “Patient” (22.6% of negative, 35.5% of positive patients) and “Private” (6.7% of negatives, 10.7% of positives). 6.1% of negative and 9.9% of positive patients reported other types of exposition, 29.3% of negative and 16.5% stated not to have had any exposition. 376 patients provided no information about any potential exposures.

**Table 3:** Expositions and travel history

Characteristics	Negative (n = 4076)		Positive (n = 121)		Total (n = 4197)		p value
Close contact to positive case							
Any exposition	2652	65.1%	104	86.0%	2756	65.7%	0.000
Colleague	1455	35.7%	47	38.8%	1502	35.8%	0.770
Patient	923	22.6%	43	35.5%	966	23.0%	0.003
Private	274	6.7%	13	10.7%	287	6.8%	0.125
Other exposition	250	6.1%	12	9.9%	262	6.2%	0.131
No exposition	1194	29.3%	20	16.5%	1214	28.9%	0.001
No information	370	9.1%	6	5.0%	376	9.0%	0.124
Recent travel abroad	195	4.8%	7	5.8%	202	4.8%	0.614
7-day incidence at travel destination (/100,000 inhabitants) (n = 189)							
Low (0–35)	81	2.0%	5	4.1%	86	2.0%	0.623
Medium (35.1–50)	57	1.4%	2	1.7%	59	1.4%	0.362
High (50.1–150)	44	1.1%	0	0.0%	44	1.0%	0.471

Expositions to SARS-COV-2-positive cases (frequency and percentage by test result), travel history (mean and inter-quartile range). p-value was obtained by univariable logistic regression (incidence reference: non-travellers)

**Fig. 1:**
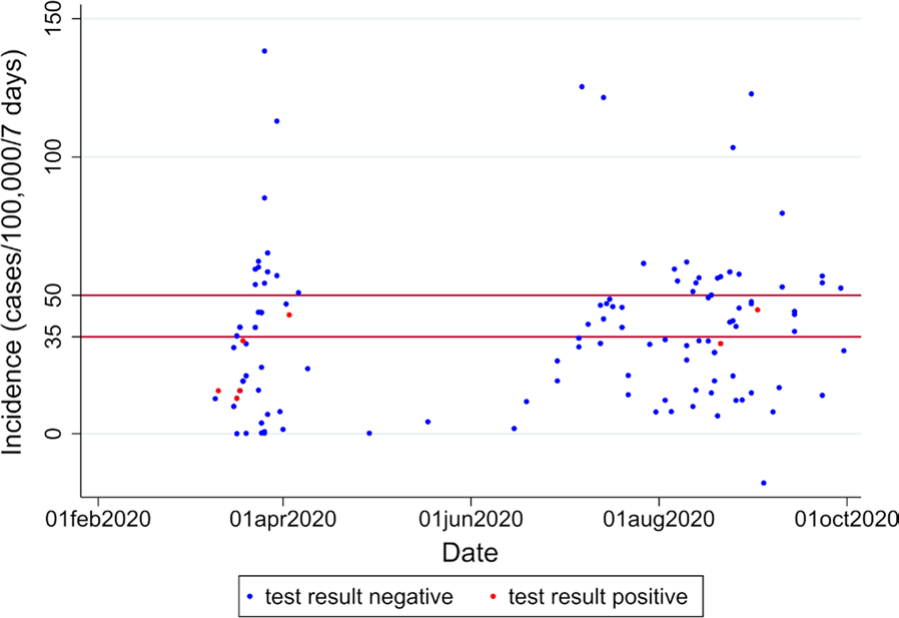
7-day incidences per 100,000 inhabitants at the travel destination of tested returnees at the time of testing. Red lines: thresholds for public health measures in Germany

195 (4.8%) of negative and 7 (5.8%) of positive patients reported a recent travel abroad. When grouping the 189 patients who provided details about their travel destination according to incidence thresholds for policy changes in Germany at the time of data collection (0–35 cases per 7 days and 100,000 inhabitants being considered low-, 35–50 intermediate and over 50 high-risk), most travellers with positive test results came from areas with incidences below the 35-threshold [16]. As being shown in Fig. 1, most imported COVID-cases were detected during the first wave in Germany.

### Univariable logistic regression analysis over time

**Fig. 2:**
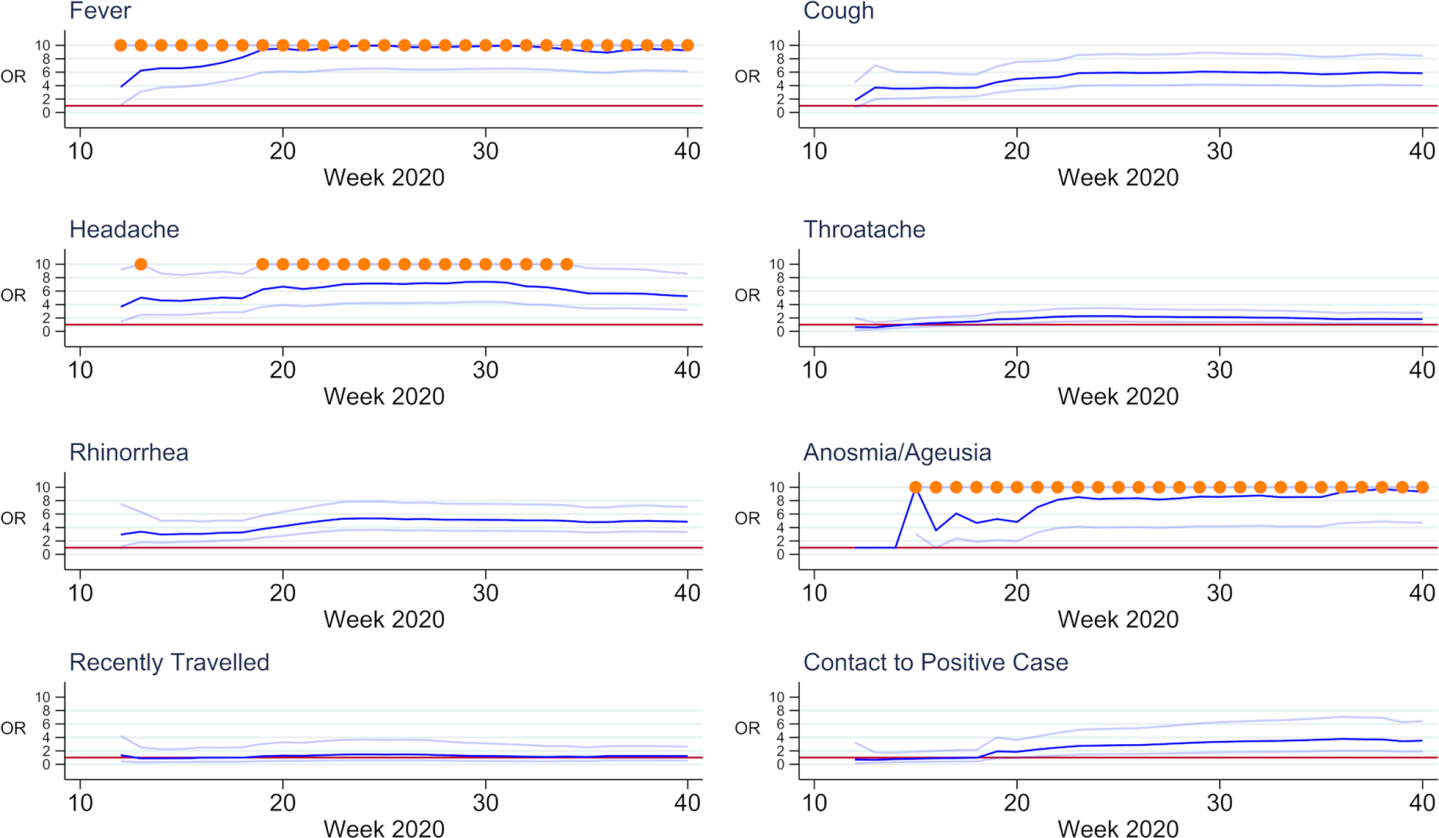
Odds ratios (dark blue) plus 95% confidence interval (light blue) in univariable logistic regression of selected variables and COVID-19 test result with weekly growing dataset. For better readability, upper confidence interval values above 10 have been truncated (orange dots)

While analysing the impact of the predictive variables in a multivariable logistic model, we noted that the OR and it’s 95% confidence interval of most variables changed over time. Figure 2 illustrates these changes on the example of selected variables with weekly growing datasets from calendar week 12 of the year 2020 on. Notably, the 95% confidence interval rapidly narrows in the first few weeks in most graphs. The graph for anosmia and/or ageusia shows viable results only from week 15 on, as it was only at this point that these symptoms were widely discussed as possible symptoms of COVID-19 and were therefore only then specifically asked for on the CRF.

### Triage scoring model

**Fig. 3:**
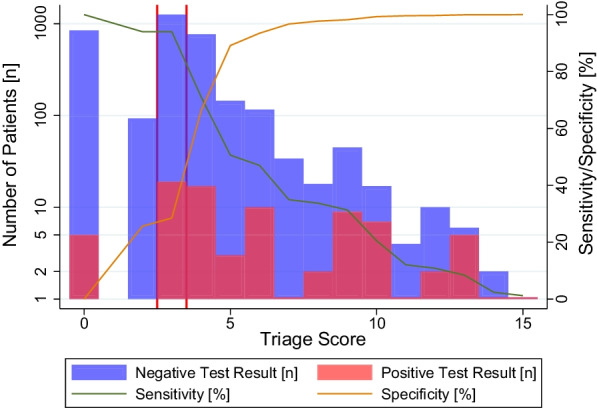
Frequency of patients with positive and negative SARS-COV2 test result by triage score (red/blue bars) and sensitivity and specificity at score cut-offs (green/orange bars). N.B.: For better readability, the frequency of patients is shown in log scale. The red lines indicate the chosen cut-off values for risk group-classification (see below)

By stepwise backwards elimination we identified several characteristics which were statistically significant at admission (see Table 4). Symptoms that could be identified as statistically significant predictors of SARS-COV-2 positivity were abdominal pain, cough, fever, anosmia and/or ageusia, and muscle pain. Our final model additionally both includes a variable for any exposition to a COVID-19 case and one specifically for contacts to a SARS-COV-2 positive patient. We then, based on the methodology described by Hartley et al. [17], calculated integer weightings for each variable by multiplying coefficients by 2 and rounding to the nearest integer, which are added together to arrive at a simplified predictive score (Table 5). We later obtained three risk groups for SARS-COV-2 positivity (low/medium/high, see Table 6) by choosing two cut-off points with the most substantial trend changes in test positivity, sensitivity and specificity while aiming for similar group sizes. Said cut-off points are shown by two vertical separator lines in Fig. 3.

**Table 4:** Variables selected for logistic regression

Characteristics	Univariable logistic regression				Multivariable logistic regression model			
	Odds ratio	p value	95% confidence interval		Odds ratio	p value	95% confidence interval	
Abdominal pain	17.56	0.000	5.99	51.51	7.38	0.070	0.85	64.36
Any exposition	3.52	0.000	1.93	6.43	3.80	0.002	1.66	8.71
Chest pain	6.84	0.000	2.81	16.69				
Cough	5.84	0.000	4.04	8.46	2.39	0.002	1.38	4.14
Diarrhea	4.04	0.023	1.21	13.50				
Otalgia	3.77	0.076	0.87	16.37				
Sputum production	8.71	0.000	3.51	21.58				
Other exposition	1.60	0.131	0.87	2.95				
Exposition to patient	1.79	0.003	1.21	2.62	1.72	0.031	1.05	2.81
Exposition to private contact	1.59	0.125	0.88	2.86				
Fatigue	5.76	0.000	3.11	10.67				
Fever	9.25	0.000	6.14	13.92	5.78	0.000	3.11	10.74
Headache	5.24	0.000	3.20	8.59				
Joint pain	15.27	0.000	7.83	29.79				
Anosmia/Ageusia	9.36	0.000	4.72	18.56	4.17	0.001	1.78	9.78
Lymphadenopathy	9.65	0.001	2.68	34.73				
Muscle pain	12.43	0.000	6.74	22.95	6.24	0.001	2.16	18.03
Nausea/Emesis	7.26	0.000	2.45	21.56				
Rhinorrhea	4.88	0.000	3.36	7.08				
Shortness of breath	2.21	0.067	0.95	5.15				
Sore throat	1.85	0.003	1.23	2.78				

Univariable analysis of clinical signs and epidemiological features associated with COVID-19 with p < 0.2 and multivariable logistic regression of selected variables

**Table 5:** Coefficients and weights of variables in derived screening score

Characteristics	Coefficients	Weights
Abdominal pain	2.00	4
Anosmia/Ageusia	1.43	3
Any exposition	1.34	3
Cough	0.87	2
Exposition to patient	0.54	1
Fever	1.75	4
Muscle pain	1.83	4

**Table 6:** Triage scores and assigned risk groups

Score	Total (n)	Positive (n)	Positive (%)	Sensitivity (%)	Specificity (%)	Assigned risk group
0	846	5	0.59	100	0	Low (n = 939, test positivity = 0.53%)
2	93	0	0.00	94	26	
3	1257	19	1.51	94	28	Medium (n = 1257, test positivity = 1.51%)
4	768	17	2.21	71	66	High (n = 1166, test positivity = 5.06%)
5	145	3	2.07	51	89	
6	116	10	8.62	47	93	
7	34	1	2.94	35	97	
8	18	2	11.11	34	98	
9	45	9	20.00	31	98	
10	17	7	41.18	20	99	
11	4	1	25.00	12	100	
12	10	2	20.00	11	100	
13	6	5	83.33	8	100	
14	2	1	50.00	2	100	
15	1	1	100.00	1	100	

SARS-COV2 Triage Scores divided into low-, medium-, and high-risk groups

The analysis of sensitivity and specificity of our final model resulted in an AUC of 77.21% (95% confidence interval (CI95%) 71.59–82.84%). We performed the internal validation of our model by subtracting the optimism obtained by bootstrapping (0.77%) from our original AUC. This yielded a final discriminative power of 76.43%.

## Discussion

The experiences at our CTU in Munich are reflecting that of pandemic response facilities that were installed at the very start of the pandemic in Europe. Once the first cases appeared in our community, our institute was able to respond with a team that was well prepared with regard to infection prevention and control measures, after having trained our team previously for outbreak situations. However, the scarcity of diagnostic capacity, the unforeseeable dynamic of the outbreak situation, and the rapidly changing state of knowledge on characteristics of the infectious agent with regard to both contagiousness and virulence, left us with no validated instrument at hand for proper triaging of individuals to be tested. Our model that we have described above provides a powerful tool that can be applied in comparable situations of scarcity of resources.

With regard to the collected clinical variables, flu-like symptoms including fever, cough and myalgia have been extensively reported elsewhere to be common signs, as has anosmia and/or ageusia as a virtually pathognomonic symptom of COVID-19 [18]. Similarly unsurprising is the effect of a close contact to SARS-COV-2 positive individuals. Although the most frequently reported exposure in our cohort was to a work colleague with COVID-infection, contact with a positive patient showed a higher correlation with COVID-19 infection in logistic regression. This correlation was shown to be so substantial that a model with an additional variable for patient contact provided the greatest power. Thus, with comparatively less knowledge and resources, health care workers seem to have been rather poorly protected against infection from patients.

### Triage score applicability

The patient data collected by us for this screening tool is based on self- or interviewer based anamnestic questions and allows for a patient stratification in situations of limited resources. At the same time the presented pre-test scoring allows for up- or down-scaling, and hence for immediate reaction to a changing balance between demand and available resources. This has been a continuous dilemma in the course of many response facilities, especially in a local point of view at the beginning of the outbreak. If, hypothetically, our testing unit would have happened to face 1.5-fold patient numbers without the immediate possibility of meeting this increase in demand and hence were forced to triage out 1681 (33.33%) patients, by applying our triage model and prioritizing patients with the highest scores we could still include 116 (92.8%) of 125 patients with subsequently positive SARS-COV2 test results.

The calculation is designed to be similarly straightforward by simply adding up the points of the applicable risk variables. Therefore, it might be more suitable than other previously published screening tools that include radiological imaging or laboratory analyses for outpatient setups with limited resources or when low pre-test probabilities do not warrant invasive diagnostics. Many of these early models were created in a context where PCR testing was still difficult or impossible to access and conventional laboratory analysis and radiological diagnostics were comparatively more readily available. It must be assumed that PCR analysis has now become so widely available that triage scores for testing are primarily of use in low-resource environments where laboratory and radiology resources are equally scarce [19].

### Further collected variables

Other collected variables, though not suitable for inclusion in our triage scoring model, may nevertheless provide information about their influence on the risk of infection:

In our cohort, groups with close patient contact (nurses, physicians, and physiotherapists) and cleaners appeared to be at the highest risk of contracting SARS-COV. These results are consistent with the findings of other researchers that physicians in particular are at disproportionate risk of infection [20, 21].

Anamnestic reported fever correlated more closely with SARS-COV-2-infection, compared to the measured temperature by the time of admission. This could be partially explained by the use of non-contact infrared thermometers, which are widely used in hospitals, airports and other public spaces and the deployment of which show advantages as well as limitations when compared to conventional thermometers [22]. Although convenient and hygienic through contactless service, their accuracy appears to be inferior in places with varying air temperature as in our outdoor admission tent. We have to take the results of the systematic temperature measurements with some degree of pre-caution, which, however, should apply in general to reports on temperature taking by using non-contact thermometers in cold environmental settings.

Similarly, travel history seemed not to be relevant for testing outcome in our cohort. The classification into risk groups by incidence at the travel destination proved insignificant in our cohort, especially as none of the high-risk travellers returned with a PCR-positive infection. After the first identified cases in Germany, which have previously been described to have been imported [23, 24], COVID-19 spread quickly throughout the country and can now be considered ubiquitous. Nevertheless, travel history is still frequently included in triage procedures.

Substantial research has been done about health risks in SARS-COV-2-positive patients with asthma compared to positive non-asthma patients [25, 26], while information about the COVID-19 infection risk of people with asthma is relatively scarce. Our findings support the assessment that people with asthma have the same or an even smaller risk of contracting COVID-19 compared to the healthy population [27], although our contribution here is weak in terms of case numbers. Parts of the immune response specific to asthma patients as well as asthma medication are being discussed as protective factors against SARS-COV-2 infection [28].

### Reliability of triage models with growing datasets

Analysis of the changes in model fit over time, as depicted in Fig. 2, can illustrate the problems researchers might face with rash analysis of limited patient data early on in an infectious disease outbreak and may explain in parts why a number of studies early in the pandemic found different and sometimes conflicting results about influences of patient characteristics on SARS-COV-2 infection. Early generated models might lack accuracy, especially in case of low numbers of outcomes paired with a high number of predictor variables [11]. Paying attention to a sufficiently large number of cases as well as further development of models over time with growing data sets should therefore be pursued. This consideration is particularly important when communicating with the general public in the communities, as public attention was geared by an expectation of perfection in epidemiological modelling [29].

### Limitations

Several limitations to this study must be acknowledged: As a very specific cohort was selected for the development of the model (mainly young, female, overall healthy individuals with some medical training), the model must be regarded as only conditionally applicable to the general population. Furthermore, due to the time restriction to the first to second wave of infections in Germany, changes caused by public measures and virus mutations in the further course of the pandemic cannot be taken into account. Moreover, growing evidence about a previously little known pathogen resulted in modifications of clinical characteristics, which in turn resulted in repetitively changed case report forms and case definitions. This led over time to an incomplete dataset. Other characteristics found by other researchers to be relevant to the risk of infection specifically in the group of HCW were not included in our questionnaire—for instance, day versus night shift workers [30]. Most characteristics, exposures and symptoms in the dataset were self-reported by patients and could therefore have been affected by various social influencing factors. Consequently, the triage model likewise does not allow for an objective assessment of individuals without their cooperation and is subject to the same limitations. As for all medical predictive models, external validation is needed further on to assess the applicability of said model.

## Conclusions

Being on a forefront of infection control and treatment, health care workers must be considered as one particularly vulnerable group in an early pandemic. To keep medical staff as well as patients safe, frequent testing has been declared a crucial tool in infection prevention and control. In situations similar to Germany in early 2020 without optimal protection from infection (through supply shortages of personal protective equipment and lack of immunity through vaccination, but also due to limited capacity and competence in HCWs in the application of principles of barrier nursing) and limited testing capacities, a triage tool that allows evidence-based pre-selection of patients can be vital for the operations of an early response facility. With this triage score, we are describing an easily accessible tool for the assessment of the probability of infection in health care workers in an outpatient setting.

Due to mutation-related changes in viral properties, further development and dissemination of diagnostic tools, discovery of additional prognostic factors, and changes in public policies, further collection of data on patient characteristics and continued development and validation of prognostic tools are necessary to tackle the biggest pandemic of the twenty-first century.

## Supplementary Information

**Additional file 1: Table S1.** Pre-existing conditions (frequency and percentage by test result), vital parameters (mean and inter-quartile range) collected in a patient subgroup. p-value was obtained by univariable logistic regression (NA: p-value calculation not applicable).

## Data Availability

The datasets generated and/or analysed during the current study are not publicly available due to conditions imposed by the ethics board, which, at the time of ethics proposal, approved of an aggregated presentation of the data only. The datasets are available from the corresponding author on reasonable request and only after permission by the involved ethics board.

## Abbreviations

SARS-COV-2: Severe acute respiratory syndrome coronavirus 2
COVID-19: Coronavirus disease 2019
RT-PCR: Real-time polymerase chain reaction
AUC: Area under the receiver operating characteristics curve
HCW: Health care workers
TRIPOD: Guidelines for transparent reporting of a multivariable prediction model for individual prognosis or diagnosis
CTU: Corona Testing Unit Munich
LMU: Ludwig-Maximilians-Universität Munich
CRF: Case report form
OR: Odds ratio
CI95%: 95% confidence interval
