# Supplementary material for: Modelling of a triage scoring tool for SARS-COV-2 PCR testing in health-care workers: data from the first German COVID-19 Testing Unit in Munich
Source: BMC Infect Dis. 2022 Aug 1;22:664. doi: 10.1186/s12879-022-07627-5 (PMC9341161; doi:10.1186/s12879-022-07627-5)
Supplement: Supplementary file 1 — Additional file 1: Table S1. Pre-existing conditions (frequency and percentage by test result), vital parameters (mean and inter-quartile range) collected in a patient subgroup. p-value was obtained by univariable logistic regression (NA: p-value calculation not applicable). [file 12879_2022_7627_MOESM1_ESM.docx]

| Characteristics | Negative (n=4076) | | Positive (n=121) | | Total (n=4197) | | p-value |
| --- | --- | --- | --- | --- | --- | --- | --- |
| Pre-existing Conditions (n=356) |  |  |  |  |  |  |  |
| Heart Disease | 8 | 2.5% | 0 | 0.0% | 8 | 2.4% | NA |
| Lung Disease | 1 | 0.3% | 0 | 0.0% | 1 | 0.3% | NA |
| Asthma | 34 | 10.7% | 0 | 0.0% | 34 | 10.0% | NA |
| Kidney Disease | 2 | 0.6% | 0 | 0.0% | 2 | 0.6% | NA |
| Nerve Disease | 2 | 0.6% | 0 | 0.0% | 2 | 0.6% | NA |
| Cancer | 6 | 1.9% | 1 | 4.3% | 7 | 2.1% | 0.440 |
| Blood Cell Disease | 4 | 1.3% | 1 | 4.3% | 5 | 1.5% | 0.270 |
| HIV | 4 | 1.3% | 0 | 0.0% | 4 | 1.2% | NA |
| Obesity | 10 | 3.2% | 2 | 8.7% | 12 | 3.6% | 0.186 |
| Diabetes | 1 | 0.3% | 0 | 0.0% | 1 | 0.3% | NA |
| Rheumatological Disease | 3 | 1.0% | 0 | 0.0% | 3 | 0.9% | NA |
| Other Disease | 66 | 20.2% | 3 | 13.0% | 69 | 19.8% | 0.407 |
| No Comorbidities | 217 | 65.2% | 18 | 78.3% | 235 | 66.0% | 0.207 |
| Vital Parameters |  | |  |  |  |  |  |
| Pulse (n=162) | 81.6 | 21.0 | 106.0 | 22.0 | 82.1 | 21.0 | 0.015 |
| Temperature (n=166) | 36.4 | 0.9 | 37.1 | 1.5 | 36.4 | 0.9 | 0.172 |
| O2 Saturation (n=159) | 96.8 | 2.0 | 93.0 | 15.0 | 96.7 | 2.0 | 0.178 |

Additional Table S1. Pre-Existing Conditions (Frequency and Percentage by Test Result), Vital Parameters (Mean and Inter-Quartile Range) collected in a patient subgroup. p-value was obtained by univariable logistic regression (NA: p-value calculation not applicable).
